# Supplementary material for: Disentangling the Taxonomic Status of Caprella penantis sensu stricto (Amphipoda: Caprellidae) Using an Integrative Approach
Source: Life (Basel). 2022 Jan 21;12(2):155. doi: 10.3390/life12020155 (PMC8878143; doi:10.3390/life12020155)
Supplement: Supplementary file 1 [file life-12-00155-s001.zip › life-1534538-supplementary/Suplementary_Material/Figure S1_Legends.pdf]

Figure S1. Maximum likelihood tree of *Caprella penantis sensu stricto* and related taxa based on mitochondrial COI sequences. Bootstrap support above 50 are indicated. Clades VA – VC are identified. The tree was rooted with *C. simia* and *C. linearis* (sequences available in GenBank: KF743437 and FJ581572, respectively). The two haplotypes (H40 and H41) from Viana do Castelo grouped in the Clade VB are highlighted in bold.
