# Supplementary material for: Disentangling the Taxonomic Status of Caprella penantis sensu stricto (Amphipoda: Caprellidae) Using an Integrative Approach
Source: Life (Basel). 2022 Jan 21;12(2):155. doi: 10.3390/life12020155 (PMC8878143; doi:10.3390/life12020155)
Supplement: Supplementary file 1 [file life-12-00155-s001.zip › life-1534538-supplementary/Suplementary_Material/Table S2.pdf]

Table S2. Morphological characters distinguishing adult males of *Caprella penantis sensu stricto* (*s.s.*) (Clade VB), Clade VA and Clade VC. Distribution locations of each clade is also provided.

	Caprella penantis s.s **(Clade VB)**	**Clade VA**	**Clade VC**
**Gnathopods 2**	Elongated with abundant setae and a larger proximal projection	Rounded with less setae and a smaller proximal projection	Rounded with some setae and a larger proximal projection
**Gills**	Small and elongated	Big and rounded	Big and rounded
• **Length / Width:**	2.5	1.5	1.5
• **Length pereonite / length gill**	2	1	1
**Distribution**	UK, Northern Spain, and Azores (Portugal)	Continental Portugal, Strait of Gibraltar, and Temara (Atlantic Morocco)	Safi (Atlantic Morocco)
