# Supplementary material for: Disentangling the Taxonomic Status of Caprella penantis sensu stricto (Amphipoda: Caprellidae) Using an Integrative Approach
Source: Life (Basel). 2022 Jan 21;12(2):155. doi: 10.3390/life12020155 (PMC8878143; doi:10.3390/life12020155)
Supplement: Supplementary file 1 [file life-12-00155-s001.zip › life-1534538-supplementary/Suplementary_Material/Table S3.pdf]

Table S3. Results of AMOVA tests comparing variation in COI sequences for each phylogenetic clade (10,100 permutations). Region definition for Clade VA: continental Portugal (Alteirinhos, Viana do Castelo, Mindelo, Labruge, Castelejo and Praia Azul) and the Strait of Gibraltar (Torreguadiaro, Tarifa Island, Puerto de Ceuta, Grottes d'Hercules, Ksar-es Seghir, Estepona, El Chorrillo, Punta Carnero, Benzú and Punta Almina); and for *Caprella penantis sensu stricto* (Clade VB): UK (Sidmouth and St. Ives), Northern Spain (Baleo, Cetarea, Oyambre), Azores (Monte da Guia) and continental Portugal (Viana do Castelo).

Species	Source of variation	d.f.	Sum of squares	Variance components	Percentage of variation	Fct	Fsc	Fst	P
Clade VA	Among regions	1	104.252	2.28487	46.49	0.46486			0.00020
	Among populations within regions	15	138.737	1.90766	38.81		0.72528		0.00000
	Within populations	63	45.523	0.72259	14.70			0.85299	0.00000
	Total	79	288.512	4.91512	100.00				
Caprella penantis sensu stricto (Clade VB)	Among regions	3	75.499	1.00479	22.30	0.22297			0.03188
	Among populations within regions	3	30.055	0.89971	19.97		0.25695		0.00000
	Within populations	58	150.907	2.60185	57.73			0.42263	0.00000
	Total	64	256.462	4.50635	100.00				
