# Supplementary material for: Disentangling the Taxonomic Status of Caprella penantis sensu stricto (Amphipoda: Caprellidae) Using an Integrative Approach
Source: Life (Basel). 2022 Jan 21;12(2):155. doi: 10.3390/life12020155 (PMC8878143; doi:10.3390/life12020155)
Supplement: Supplementary file 1 [file life-12-00155-s001.zip › life-1534538-supplementary/Suplementary_Material/Table S4.pdf]

Table S4. Pairwise Fst values between locations for each phylogenetic clade, based on 537 bp of mitochondrial cytochrome c oxidase subunit I gene. Only locations with three or more sequences were used. Significant values for P < 0.05, obtained through 10,000 permutations, are depicted in bold. Negative values not shown (n.s.).

Clade VA

REGION	LOCALITION	V. Castelo	Alteirinhos	P. Azul	Castelejo	Labruge	Mindelo	P. Almina	Benzú	P. Carnero	El Chorrillo	Estepona	G. d´Hercules	K. Seghir	P. Ceuta	Tarifa I.	Tarifa I. 2	Torreguadiaro
Continental Portugal	Viana do Castelo	0.00000																
	Alteirinhos	**0.78661**	0.00000															
	Praia Azul	**0.83266**	**0.91847**	0.00000														
	Castelejo	**0.75408**	n.s.	**1.00000**	0.00000													
	Labruge	**0.50638**	**0.89933**	0.92857	**0.91489**	0.00000												
	Mindelo	**0.38829**	**0.88931**	**0.92771**	**0.90909**	0.37615	0.00000											
Strait of Gibraltar	Punta Almina	**0.81267**	**0.90090**	**0.89427**	**0.88235**	**0.84049**	**0.84906**	0.00000										
	Benzú	**0.86196**	**0.95198**	**1.00000**	**1.00000**	**0.94805**	**0.94737**	0.00000	0.00000									
	Punta Carnero	**0.82959**	**0.89071**	0.87879	**0.86441**	0.83784	**0.85401**	**0.37615**	**0.57895**	0.00000								
	El Chorrillo	**0.84223**	**0.93728**	0.97059	**0.96787**	0.90625	**0.91201**	n.s.	0.11111	0.44444	0.00000							
	Estepona	**0.88776**	**0.96219**	1.00000	**1.00000**	0.95000	**0.95219**	**0.70732**	**1.00000**	0.76471	0.90000	0.00000						
	Grottes d´Hercules	**0.74117**	**0.79128**	0.79167	**0.72414**	0.75000	**0.76938**	**0.73611**	**0.84906**	0.66667	0.78571	0.86111	0.00000					
	Ksar-es Seghir	**0.78243**	**0.83388**	0.78125	**0.74888**	0.73000	**0.77542**	**0.75202**	**0.83673**	0.68571	0.77778	0.82500	0.21739	0.00000				
	Puerto de Ceuta	**0.84213**	**0.92845**	**0.94805**	**0.94118**	**0.89522**	**0.90000**	0.00000	0.00000	**0.45946**	n.s.	**0.83099**	**0.79396**	**0.78827**	0.00000			
	Tarifa Island	**0.75084**	**0.76265**	**0.72450**	**0.67092**	**0.72277**	**0.72923**	**0.29160**	**0.31903**	n.s.	**0.26899**	**0.55630**	**0.57721**	**0.64055**	**0.30887**	0.00000		
	Tarifa Island 2	**0.86564**	**0.94115**	1.00000	**1.00000**	0.94118	**0.94313**	**0.70732**	**1.00000**	0.42857	0.90000	1.00000	0.79167	0.78125	**0.83099**	0.17523	0.00000	
	Torreguadiaro	**0.89492**	**0.95811**	**0.98127**	**0.97962**	**0.94606**	**0.94815**	**0.73721**	**0.92895**	**0.79603**	**0.8607**	n.s.	**0.8846**	**0.86147**	**0.82115**	**0.61131**	**0.95723**	0.00000

Caprella penantis sensu stricto (Clade VB)

REGIONS	LOCALITION	Sidmouth	St. Ives	Baleo	Cetarea	Oyambre	M. Guia	V. Castelo
UK	Sidmouth	0.00000						
	St. Ives	**0.27605**	0.00000					
Northern Spain	Baleo	**0.70614**	**0.31587**	0.00000				
	Cetarea	**0.70376**	**0.29935**	**0.62791**	0.00000			
	Oyambre	**0.60551**	**0.32001**	**0.43674**	0.15544	0.00000		
Azores	Monte da Guia	**0.49462**	**0.36487**	**0.25916**	**0.37500**	**0.36770**	0.00000	
Continental Portugal	Viana do Castelo	**0.68633**	**0.27836**	**0.35135**	0.45000	**0.36881**	**0.24696**	0.00000
