# Supplementary figures and images for: Disentangling the Taxonomic Status of Caprella penantis sensu stricto (Amphipoda: Caprellidae) Using an Integrative Approach
Source: Life (Basel). 2022 Jan 21;12(2):155. doi: 10.3390/life12020155 (PMC8878143; doi:10.3390/life12020155)

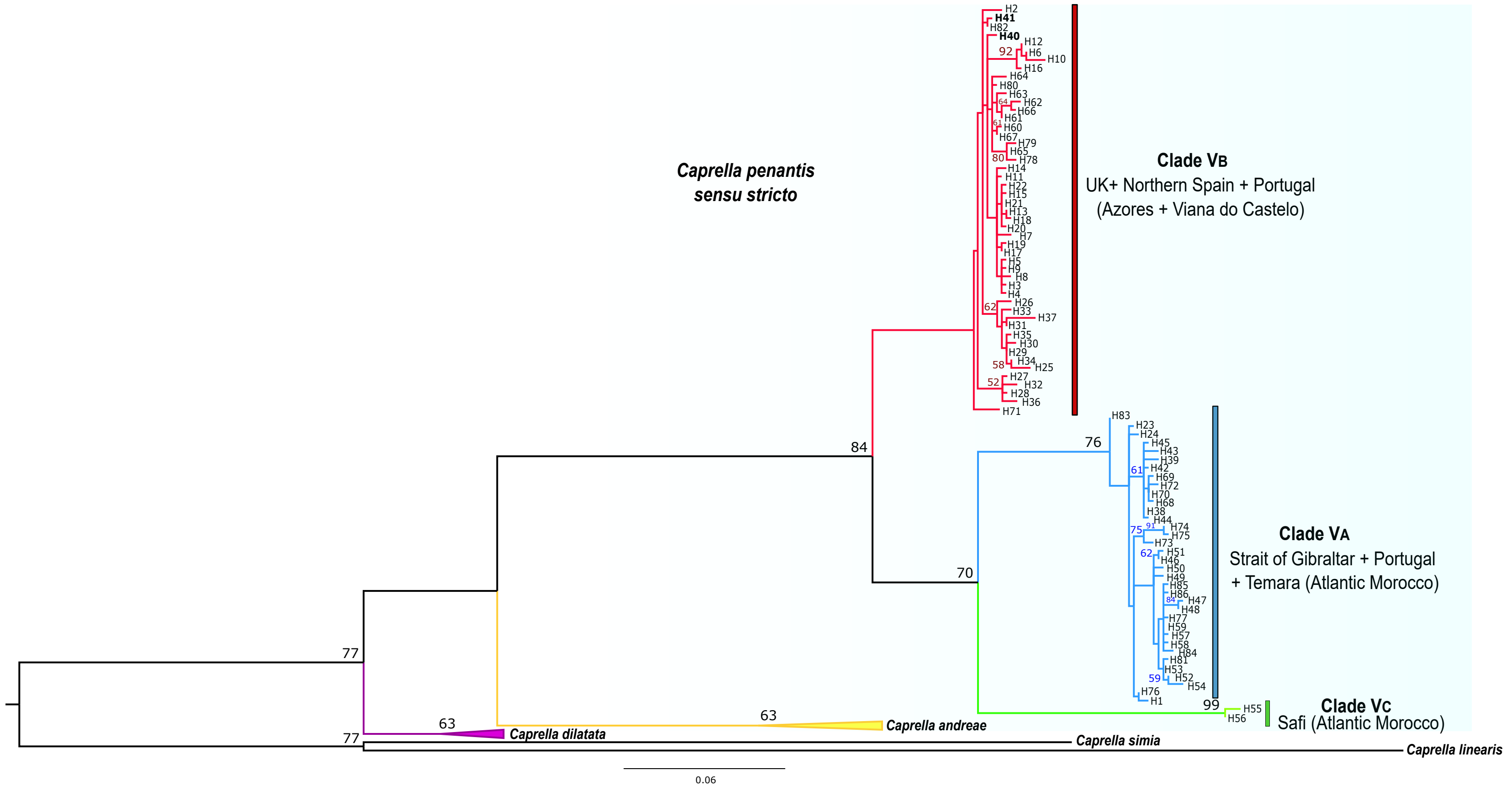

Supplement: Supplementary file 1 [file life-12-00155-s001.zip › life-1534538-supplementary/Suplementary_Material/Figure S1.pdf]
